# Release and uptake mechanisms of vesicular Ca^2+^ stores

**DOI:** 10.1007/s13238-018-0523-x

**Published:** 2018-03-16

**Authors:** Junsheng Yang, Zhuangzhuang Zhao, Mingxue Gu, Xinghua Feng, Haoxing Xu

**Affiliations:** 1 Collaborative Innovation Center of Yangtze River Delta Region Green Pharmaceuticals, College of Pharmaceutical Sciences, Zhejiang University of Technology, Hangzhou, 310014 China; 2 The Department of Molecular, Cellular, and Developmental Biology, University of Michigan, Ann Arbor, MI 48109 USA

**Keywords:** Ca^2+^ stores, lysosomes, vesicles, refilling, organelle membrane contact sites (MCSs)

## Abstract

Cells utilize calcium ions (Ca^2+^) to signal almost all aspects of cellular life, ranging from cell proliferation to cell death, in a spatially and temporally regulated manner. A key aspect of this regulation is the compartmentalization of Ca^2+^ in various cytoplasmic organelles that act as intracellular Ca^2+^ stores. Whereas Ca^2+^ release from the large-volume Ca^2+^ stores, such as the endoplasmic reticulum (ER) and Golgi apparatus, are preferred for signal transduction, Ca^2+^ release from the small-volume individual vesicular stores that are dispersed throughout the cell, such as lysosomes, may be more useful in local regulation, such as membrane fusion and individualized vesicular movements. Conceivably, these two types of Ca^2+^ stores may be established, maintained or refilled via distinct mechanisms. ER stores are refilled through sustained Ca^2+^ influx at ER-plasma membrane (PM) membrane contact sites (MCSs). In this review, we discuss the release and refilling mechanisms of intracellular small vesicular Ca^2+^ stores, with a special focus on lysosomes. Recent imaging studies of Ca^2+^ release and organelle MCSs suggest that Ca^2+^ exchange may occur between two types of stores, such that the small stores acquire Ca^2+^ from the large stores via ER-vesicle MCSs. Hence vesicular stores like lysosomes may be viewed as secondary Ca^2+^ stores in the cell.

## Introduction

Ca^2+^ is a common second messenger in the cell that has been implicated in the regulation of virtually all aspects of cellular life, including cell growth, differentiation, motility and death (Clapham, 2007; Berridge, 2012). Upon binding to its effector proteins, such as calmodulin and synaptotagmins, Ca^2+^ regulates a variety of cellular processes, such as gene transcription, secretion and muscle contraction (Clapham, 2007; Berridge, 2012).

**Figure 1:**
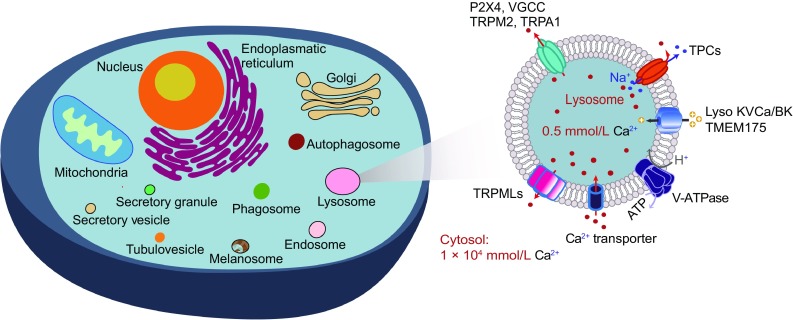
**Intracellular Ca**^**2+**^ **stores**. Diagram of intracellular Ca^2+^ stores, illustrating Ca^2+^ release and uptake mechanisms. Large, continuous stores include the ER, the Golgi apparatus, mitochondria and the nucleus. Small, non-continuous vesicular stores, include endosomes, lysosomes, (auto)phagosomes and secretory vesicles, as well as vesicles in specialized cell types, such as tubulovesicles in parietal cells, melanosomes in melanocytes, synaptic vesicles in neurons and secretory granules in neurosecretory cells. TRPMLs, TPCs, P2X4, VGCCs, TRPA1 and TRPM2 are potential Ca^2+^ release channels in lysosomes. The H^+^ gradient in the lysosome is established and maintained by V-ATPases, and the Ca^2+^ gradient in lysosomes is established and maintained by a putative Ca^2+^ transporter/channel

Ca^2+^ signaling is switched on and off via transient changes in cytosolic Ca^2+^ concentration ([Ca^2+^]_cyt_), either locally or globally. Under resting conditions, [Ca^2+^]_cyt_ is low (~100 nmol/L), while [Ca^2+^]s are at least several thousand fold higher in the extracellular environment (~2 mmol/L) and in the lumen of organelles, such as the endoplasmic reticulum (ER, 0.3–0.7 mmol/L) and lysosomes (0.4–0.6 mmol/L), which serve as intracellular Ca^2+^ stores (Berridge et al., 2000; Morgan et al., 2011; Pizzo et al., 2011; Bengtson and Bading, 2012) (Fig. 1). Upon stimulation by extracellular transmitters and hormones, activation of Ca^2+^ influx channels in the plasma membrane (PM) or Ca^2+^ release channels in the ER, such as inositol 1,4,5-triphosphate receptors (IP3Rs) and ryanodine receptors (RyRs) (Clapham, 2007; Berridge, 2012), leads to rapid [Ca^2+^]_cyt_ increases of 10–100 fold to μmol/L concentrations. Upon termination of Ca^2+^ signaling, [Ca^2+^]_cyt_ returns quickly to a resting level, via primary, principally sarcoendoplasmic reticulum calcium transport ATPase (SERCA), pumps and secondary Ca^2+^ transporters in the PM and membranes of intracellular Ca^2+^-sequestering organelles (Clapham, 2007; Berridge, 2012).

The ER, which consists of interconnected and continuous tubules and cisternae and constitutes the largest membrane-bound organelle in the cell, is the most important intracellular Ca^2+^ storage site in the cell (Prakriya and Lewis, 2015; Phillips and Voeltz, 2016). Dys-regulation of intracellular Ca^2+^ homeostasis, either during signal initiation or termination, is associated with a number of genetic diseases (Berridge, 2012).

In addition to the ER, the Golgi apparatus, nucleus, and mitochondria also store Ca^2+^ (Rizzuto et al., 2012; Patel and Cai, 2015; Xu et al., 2015; Raffaello et al., 2016; Bagur and Hajnoczky, 2017) (Fig. 1). These membrane-bound organelles are interconnected and at least partially continuous in their lumens, providing a large storage capacity (Prakriya and Lewis, 2015; Phillips and Voeltz, 2016). The release and uptake mechanisms for these large stores have been studied and reviewed extensively (Prakriya and Lewis, 2015).

In contrast, intracellular vesicles, of which there are tens to hundreds in a cell, are a much less understood Ca^2+^ store organelles. Acidic stores (e.g., endosomes, lysosomes, secretory granules and lysosome-related organelles) can also undergo regulated Ca^2+^ release (Morgan et al., 2011) (Fig. 1). Compared with the large Ca^2+^ stores, vesicular Ca^2+^ storage and release has been technically challenging to study due to the relatively small signal amplitude that can be generated by the small-sized releasable Ca^2+^ pool in individual vesicles (Morgan et al., 2015; Xu et al., 2015). Fortunately, this roadblock has been partially removed with the recent development of organelle-targeted genetically-encoded Ca^2+^ indicators (GECIs) (Shen et al., 2012; Morgan et al., 2015; Xu et al., 2015; Garrity et al., 2016; Sahoo et al., 2017). Lysosomes are the cell’s recycling centers, playing essential roles in the basic cell biological processes of endocytosis, exocytosis and autophagy (Morgan et al., 2011; Patel and Cai, 2015; Xu and Ren, 2015).

In this review, we focus our discussion on the Ca^2+^ release and refilling mechanisms of the lysosome. We summarize the evidence supporting each of two distinct uptake hypotheses: the long known pH-dependent Ca^2+^ uptake hypothesis (Christensen et al., 2002; Morgan et al., 2011) and the recently introduced ER-dependent refilling hypothesis (Garrity et al., 2016; Wang et al., 2017). We will then extend the discussion to other vesicular Ca^2+^ stores in the cell, including cell-type-specific vesicles.

## Large Ca^2+^ stores

The ER, which is the largest intracellular Ca^2+^ store in the cell (Phillips and Voeltz, 2016), has a luminal Ca^2+^ concentration ([Ca^2+^]_ER_) that is about 5,000 times higher than resting [Ca^2+^]_cyt_ (Berridge et al., 2000; Prakriya and Lewis, 2015). SERCA pumps establish and maintain this very high [Ca^2+^]_ER_. Upon stimulation, ER releases Ca^2+^ into the cytoplasm via IP3Rs and RyRs (Prakriya and Lewis, 2015). A single stimulation event from an extracellular cue may only result in incomplete depletion of the store (Berridge et al., 2000; Prakriya and Lewis, 2015). Nevertheless, given the large volume of the ER’s interconnected tubules, substantial increases in global [Ca^2+^]_cyt_ can be achieved with each stimulation event (Prakriya and Lewis, 2015), triggering various signal transduction cascades in the cell. In muscle cells, the opening of RyRs in the sarcoplasmic reticulum, a specialized type of ER in striated muscle cells, produces the massive increases in [Ca^2+^]_cyt_ required for muscle contraction.

ER stores can be refilled by the well characterized process of store-operated Ca^2+^ entry (SOCE). SOCE relies on the collaborative actions of stromal interaction molecule (STIM) proteins, which serve as ER luminal Ca^2+^ sensors, and Orai proteins, which act as store-operated Ca^2+^ channels in the PM. Upon ER Ca^2+^ store depletion, STIM1 and STIM2 become activated and oligomerized (Prakriya and Lewis, 2015), favoring the formation of membrane contact sites (MCSs) between ER tubules and the PM (Stathopulos and Ikura, 2017). Orai proteins in the PM then accumulate through diffusion to the PM side of MCSs (Berridge et al., 2000; Prakriya and Lewis, 2015; Phillips and Voeltz, 2016). SOCE is triggered when STIM proteins bind directly to and thereby activate Orai channels, resulting in a sustained Ca^2+^ influx from the extracellular space that raises local [Ca^2+^]_cyt_ in ER-PM MCS locations (Prakriya and Lewis, 2015). The imported Ca^2+^ is then taken up into the ER, resulting in ER Ca^2+^ store refilling, via high-affinity (low μmol/L range) SERCA pumps (Clapham, 2007). Detailed descriptions of the molecular mechanisms of ER Ca^2+^ channels and SOCE can be found in several recently-published excellent reviews (Prakriya and Lewis, 2015; Lopez et al., 2016; Putney et al., 2017; Stathopulos and Ikura, 2017).

Other intracellular organelles, including the nucleus, Golgi apparatus and mitochondria, serve as large Ca^2+^ stores. The nuclear envelope is continuous with ER membranes and contains IP3R and RyR Ca^2+^ release channels as well as SERCA Ca^2+^ uptake transporters (Bootman et al., 2009). SERCA, IP3Rs and RyRs are also expressed in Golgi apparatus membranes, which are partially interconnected but hold unevenly-distributed Ca^2+^ stores, ranging from ~130 μmol/L in the trans-Golgi cisterna to 250 μmol/L in the cis-Golgi cisterna (Pizzo et al., 2011). Besides SERCA pump-mediated Ca^2+^ uptake, Ca^2+^ can also be brought into the Golgi apparatus by way of secretory pathway Ca^2+^-ATPases (Pizzo et al., 2011). Finally, mitochondria are known to uptake cytosolic Ca^2+^ into their matrix under high [Ca^2+^]_cyt_ conditions, making them, in essence, a cellular Ca^2+^ sink (De Stefani et al., 2016). Mitochondrial Ca^2+^ uptake is driven by a large negative membrane potential (∆ψ) in the inner membrane and mediated by voltage-dependent anion channels (VDACs) in the outer membrane and mitochondrial Ca^2+^ uniporters in the inner membrane (De Stefani et al., 2016).

Because of their collectively large luminal volumes, the ER, nucleus, Golgi apparatus and mitochondria function as the large Ca^2+^ stores of the cell. Mobilizing and emptying these large stores would result in substantial increases in [Ca^2+^]_cyt_, which in theory are preferentially suited for signal transduction.

## Small Ca^2+^ stores

Lysosomes are acidic membrane-bound organelles responsible for degrading macromolecules from both intracellular and extracellular sources (Xu and Ren, 2015). Early studies detected Ca^2+^ release induced by nicotinic acid adenine dinucleotide phosphate (NAADP) in non-ER Ca^2+^ stores (Lee and Aarhus, 1995; Calcraft et al., 2009; Morgan et al., 2011). Glycyl-L-phenylalanine-naphthylamide (GPN), a di-peptide that is degraded in lysosomes by luminal cathepsins, induces Ca^2+^ release if applied alone and abolishes NAADP-induced Ca^2+^ release via osmotic swelling of lysosomes, giving rise to the notion that lysosomes can act as NAADP-targeted Ca^2+^ stores (Morgan et al., 2011). Calibration experiments employing lysosome-targeted, pH-corrected luminal Ca^2+^ indicators (e.g., Fura-Dextran dyes) have indicated that the Ca^2+^ concentration in the lysosome lumen ([Ca^2+^]_Ly_) is about 0.5 mmol/L, which is comparable to [Ca^2+^]_ER_ (Christensen et al., 2002; Lloyd-Evans et al., 2008). Ca^2+^ release from individual lysosomes is limited by their small volume (typically <0.3 μm in diameter). Hence, lysosomes and other acidic stores, such as secretory granules, are referred to as small Ca^2+^ stores.

## Ca^2+^ release channels of the lysosome

The recognition of lysosomes as intracellular Ca^2+^ stores and the potential roles of lysosomal Ca^2+^ release in regulating lysosomal membrane fusion and fission have prompted intense investigation of lysosomal Ca^2+^ release pathways (Patel and Cai, 2015). Using lysosome-targeted GECIs (Shen et al., 2012; Morgan et al., 2015), together with the recently-developed whole-lysosome patch-clamp technique, several candidate release channels have been identified, along with the cellular cues that activate them (Calcraft et al., 2009; Wang et al., 2012; Cang et al., 2013; Cao et al., 2015a, b; Xu and Ren, 2015). These cellular cues may serve as mobilizers of the lysosomal Ca^2+^ stores, similar to IP3R signaling in the ER.

### Mucolipin subfamily of transient receptor potential (TRPML) channels

The TRPML channels, which consist of TRPML1, TRPML2 and TRPML3 (a.k.a. MCOLN1–3), are Ca^2+^-permeable cation channels expressed in endosome and lysosome membranes (Xu and Ren, 2015; Xiong and Zhu, 2016; Grimm et al., 2017) (Fig. 1). TRPML1, which is widely expressed in most cell types, is localized predominantly to late endosomes and lysosomes (Cheng et al., 2010). TRPML2 and TRPML3 are also localized to early and recycling endosomes in addition to late endosomes and lysosomes (Cheng et al., 2010). TRPML-mediated Ca^2+^ release may regulate Ca^2+^-dependent lysosomal membrane trafficking events involved in a variety of basic cell biological processes, including lysosomal exocytosis, autophagy and membrane repair (Xu and Ren, 2015; Xiong and Zhu, 2016; Grimm et al., 2017). In humans, loss-of-function mutations of TRPML1 cause type IV mucolipidosis (ML-IV), a lysosomal storage disease (LSD).

Phosphatidylinositol 3,5-bisphosphate (PI(3,5)P_2_), an endolysosome-specific phosphoinositide, may serve as an endogenous TRPML agonist (Dong et al., 2010a, b). Reactive oxygen species have been shown to activate TRPML1 directly, triggering Ca^2+^ release and Ca^2+^-dependent lysosome biogenesis and autophagy (Zhang et al., 2016). Furthermore, mucolipin-specific synthetic agonists (ML-SAs) have been identified and shown to regulate various TRPML-dependent lysosomal functions by mimicking endogenous agonists (Shen et al., 2012; Xu and Ren, 2015; Grimm et al., 2017). Recent cryo-electron microscope structural images of TRPML1 and TRPML3 revealed that ML-SA1 binds to residues in the S5 and S6 helices of these TRPMLs (Schmiege et al., 2017; Zhou et al., 2017), which form an activation gate. Hence, cellular cues, or synthetic agonists, can induce lysosomal Ca^2+^ release via direct binding to TRPML channels. Furthermore, because TRPML currents are strongly rectifying at the inward direction, cellular cues can also regulate TRPML-mediated Ca^2+^ release by modulating lysosomal ∆ψ a driving force for Ca^2+^ release (Cheng et al., 2010; Dong et al., 2010a, b). Indeed, recently identified lysosome Na^+^ and K^+^ channels that regulate lysosome ∆ψ were shown to modulate the Ca^2+^ release from TRPML1 (Cao et al., 2015a, b; Xu and Ren, 2015; Xiong and Zhu, 2016; Wang et al., 2017).

### Two-pore channels (TPC) channels

TPC1 and TPC2 channels, encoded by *TPCN1* and *TPCN2,* respectively, are localized on endosomal and lysosomal membranes (Calcraft et al., 2009) (Fig. 1). Both TPC1 and TPC2 are ubiquitously expressed in mammalian cells. Whole-lysosome patch-clamping studies suggested that mammalian TPCs are Na^+^-selective with limited Ca^2+^ permeability (Wang et al., 2012; Cang et al., 2013). However, studies from multiple laboratories reported that TPC overexpression promoted lysosomal Ca^2+^ release (Brailoiu et al., 2009; Calcraft et al., 2009; Pitt et al., 2010; Ruas et al., 2010; Grimm et al., 2017), suggesting that the relatively small Ca^2+^ permeability of TPCs is physiologically significant.

NAADP (in nmol/L ranges) is the most potent Ca^2+^-mobilizing second messenger regulating intracellular Ca^2+^ stores (Lee and Aarhus, 1995) and TPCs are, thus far, the most promising candidate receptors for NAADP (Brailoiu et al., 2009; Calcraft et al., 2009; Pitt et al., 2010; Ruas et al., 2010; Grimm et al., 2017). However, radiolabeled NAADP has also been reported to bind other unidentified proteins in TPC knockout cells (Lin-Moshier et al., 2012; Walseth et al., 2012). Hence, resolving how Na^+^-selective TPCs are involved in NAADP-induced lysosomal Ca^2+^ release will require further investigation. On the other hand, PI(3,5)P_2_ can also activate whole-lysosome TPC currents (Wang et al., 2012; Xu and Ren, 2015). The endogenous protein kinase C inhibitor sphingosine has been reported to induce TPC1-dependent lysosomal Ca^2+^ release (Hoglinger et al., 2015). However, whether sphingosine activates TPCs directly has yet to be confirmed with direct whole-lysosome recording. The relative contributions of TRPMLs and TPCs in PI(3,5)P_2_-induced lysosomal Ca^2+^ release remain to be established (Wang et al., 2012; Xu and Ren, 2015).

### Other lysosomal Ca^2+^ channels

P2X4 (purigenic receptor X4), an ATP-gated cation channel first discovered in the PM of various cell types, also resides on the lysosomal membranes of Cos1 cells where it can be activated by luminal ATP and alkalization (Qureshi et al., 2007; Huang et al., 2014) (Fig. 1). Ca^2+^ release through lysosomal P2X4 has been implicated in lysosomal membrane fusion in a calmodulin-dependent manner (Cao et al., 2015a, b). It is not clear whether P2X4 is expressed ubiquitously in mammalian lysosomes, or restrictively in certain cell types, as has been reported in tissue distribution studies (Qureshi et al., 2007).

TRPA1 (transient receptor potential ankyrin 1) is a Ca^2+^-permeable non-selective cation channel in somatosensory neurons that is activated by plant-derived chemicals, such as allyl isothiocyanate (a major ingredient of mustard oil) (Jordt et al., 2004). Recently, it was reported that TRPA1 is also expressed on peripheral lysosomes in somatosensory neurons, where it mediates allyl isothiocyanate-induced dense-core vesicle exocytosis and neuropeptide release (Shang et al., 2016).

TRPM2, a Ca^2+^ permeable non-selective cation channel gated by ADP ribose and Ca^2+^, is expressed on the PMs of neurons, pancreatic cells, and immune cells (Lange et al., 2009). TRPM2 is also localized on lysosomes in pancreatic β cells, leading to the proposition that TRPM2-mediated lysosomal Ca^2+^ release may regulate insulin secretion (Lange et al., 2009).

In central nervous system neurons, P/Q-type voltage-gated Ca^2+^ channels (VGCCs), encoded by *CACNA1*, mediate the Ca^2+^ entry that triggers neurotransmitter release. In a recent study, Tian et al., found that the α1A subunit of VGCCs is also present on lysosomal membranes in both fruit flies and mice, and is required for autophagosome-lysosome fusion (Tian et al., 2015).

In summary, both ubiquitous and cell-type-specific lysosomal expression of Ca^2+^ release channels have been described. They are activated by diverse cellular cues. Some are lysosome-committed channels, while others are dually expressed on lysosomal membranes and PMs.

### Ca^2+^-dependent membrane trafficking of individual lysosomes

Generally, mammalian cells each have several hundred lysosomes, which are heterogeneous in size and morphology, as well as in their ionic and lipid compositions (Xu and Ren, 2015). Under physiological conditions, lysosomal Ca^2+^ channel-activating cellular signals are likely only present in a subset of lysosomes. Hence, lysosomal Ca^2+^ release from individual lysosomes may not be synchronized in a manner that gives rise to global increases in [Ca^2+^]_cyt_. However, such localized Ca^2+^ release may be sufficient to regulate local membrane trafficking events, such as fusion and fission (Xu and Ren, 2015). Theoretically, the decision to fuse vesicles should be determined based on the luminal cargo contents of individual vesicles (Xu and Ren, 2015). Hence, under physiological conditions in intact cells, lysosomal Ca^2+^ release is likely conducted by individual lysosomes depending on need. Notwithstanding, in some experimental settings, synchronized lysosomal Ca^2+^ release may be amplified by ER Ca^2+^ release triggering further cell signaling transduction (Kilpatrick et al., 2016a, b); it is not known whether such Ca^2+^-induced Ca^2+^ release occurs under physiological conditions.

## Possible H^+^-dependent Ca^2+^ uptake mechanisms in the lysosome

The mechanisms that establish and maintain the massive 5,000-fold Ca^2+^ concentration gradient across the lysosomal membrane are of great interest. The prevailing view in the literature is that the lysosomal H^+^ gradient is essential for lysosomal Ca^2+^ store maintenance and refilling. Using both cytosolic and luminal Ca^2+^ dyes, researchers have shown that manipulations that cause lysosomal pH dissipation, such as V-ATPase inhibition, lead to lysosomal Ca^2+^ release, while restoration of the acidic luminal pH is accompanied Ca^2+^ store replenishment (Christensen et al., 2002; Lloyd-Evans et al., 2008; Calcraft et al., 2009; Dickson et al., 2012; Shen et al., 2012). Hence, it was proposed that a Ca^2+^/H^+^ exchanger (CAX) may drive pH-dependent Ca^2+^ uptake into lysosomes (Christensen et al., 2002; Morgan et al., 2011). CAXs are well known for their expression on vacuoles (lysosome-like organelles in yeast and plants) (Pittman, 2011). CAXs were long thought to be absent from metazoans. Although CAX genes have been identified more recently in some echinoderm, mollusk, fish, amphibian and non-placental mammal species, they have not been found in placental mammals thus far (Melchionda et al., 2016), suggesting that they might not be responsible for lysosomal Ca^2+^ uptake, at least not in placental mammals (Patel and Docampo, 2010). It is also possible that pH gradients may drive Ca^2+^ uptake indirectly in the absences of CAXs, such as through Na^+^/H^+^ exchangers and Na^+^/Ca^2+^ exchangers in series. That being said, Na^+^/H^+^ exchangers have thus far been demonstrated to be expressed in endosomes, but not lysosomes (Morgan et al., 2011).

The fact that CAXs have been remained putative in placental mammals for more than a decade encourages a revisiting of the evidence that led to the pH hypothesis. Notably, all presumed lysosomal Ca^2+^-mobilizing agents in earlier studies (i.e., GPN, Baf-A1, and NAADP) also cause lysosomal H^+^ release (Yoshimori et al., 1991; Morgan and Galione, 2007; Scott and Gruenberg, 2011; Appelqvist et al., 2012). Due to the pH sensitivities of most cytosolic Ca^2+^ dyes and probes (Rudolf et al., 2003), the presumed Ca^2+^ signals in the earlier studies may have contained, in some portion, pH signals or other unidentified pH-mediated non-Ca^2+^-dependent signals. Indeed, GPN-induced or Baf-A1-induced Ca^2+^ signals were found to be remain largely intact in the presence of a potent intracellular Ca^2+^ chelator, namely BAPTA-AM [1,2-bis(2-aminophenoxy)ethane-N,N,N′,N′-tetraacetic acid tetrakis(acetoxymethyl ester)], in a recent study (Garrity et al., 2016). In other words, the observed sensitivity to V-ATPase blockers in earlier lysosomal Ca^2+^ content studies might have contained a contaminating pH component. Hence, it is possible that secondary changes in Ca^2+^ buffering and ionic composition in the lysosome lumen consequent to pH changes may have led to a misinterpretation of previous data (Dickson et al., 2012; Garrity et al., 2016).

### *In situ* assay of lysosomal Ca^2+^ refilling

The low pH environment in the lysosome lumen makes it challenging to measure and monitor [Ca^2+^]_LY_ accurately. Thus, there has been a need for an assay that can detect Ca^2+^ release and refilling without interfering with lysosomal pH. To monitor lysosomal Ca^2+^ release, Shen et al. (Shen et al., 2012) fused a GECI (i.e., GCaMP) to the cytosolic N-terminus of TRPML1. Using this lysosome-targeted GECI, Garrity et al. (Garrity et al., 2016) developed a robust lysosomal Ca^2+^ refilling assay, in which consecutive applications of ML-SAs trigger consecutive bouts of Ca^2+^ release at 5-min intervals. The initial application of ML-SA depletes lysosomal Ca^2+^ stores, such that the response to the second application depends on lysosomal Ca^2+^ refilling during the 5-min interval (Garrity et al., 2016). To rule out the possibility that the GECI also detects ML-SA1-induced Ca^2+^ responses from organelles other than endolysosomes, control experiments were performed in which lysosomal Ca^2+^ stores were depleted with GPN. Importantly, a TRPML1 agonist, whose specificity was confirmed with mouse knockouts, was shown to be cell permeable with reversible effects (Shen et al., 2012; Garrity et al., 2016). In addition, the specificity of ML-SAs ensures that one is observing real changes in lysosomal Ca^2+^, as opposed to pH changes associated with other Ca^2+^-mobilizing reagents acting on lysosomes (Garrity et al., 2016). Indeed, ML-SA1-induced responses were completely abolished by BAPTA-AM treatment, consistent with Ca^2+^-specificity of the signal (Garrity et al., 2016). In contrast, GPN- or Baf-A1-induced presumed-to-be-Ca^2+^-specific responses persisted in the presence of BAPTA-AM (Garrity et al., 2016). Therefore, these results suggest that although GPN mobilizes more than just Ca^2+^, it is a very specific lysosome-disrupting agent. Hence, it can be used as a powerful tool to confirm the lysosome-specificity of other Ca^2+^-mobilizing reagents, such as ML-SA1.

The aforementioned lysosome-targeted GCaMP and ML-SA assay is the first robust and sensitive method developed with the ability to measure lysosomal Ca^2+^ release directly, independent of intracellular pH. It allows for time-lapse examination of lysosomal Ca^2+^ store depletion and refilling with acute (<5 min) application of various pharmacological reagents, which has many advantages, including amenability to prolonged treatment protocols, as have been used predominantly in previous studies.

Using this powerful refilling assay, Garrity et al., found that dissipation of the proton gradient in the lysosome (e.g., by V-ATPase inhibitors) has little to no impact on naïve Ca^2+^ stores or their refilling (2016). This result is inconsistent with predictions based on the prevailing pH-dependent hypothesis and, instead, suggests that lysosomal Ca^2+^ refilling is likely to be pH independent. It is possible that V-ATPase blockers in earlier studies (e.g., (Christensen et al., 2002; Calcraft et al., 2009; Morgan et al., 2011)) abolished the indirect effects of lysosomal H^+^ release on pH-sensitive Ca^2+^ dyes and probes (2016).

## ER Ca^2+^ and IP3Rs are required for lysosomal Ca^2+^ refilling

In sharp contrast to the lack of V-ATPase effects, depletion of ER Ca^2+^ stores by SERCA inhibitors was shown to abolish lysosomal Ca^2+^ refilling (2016). Furthermore, inhibition of IP3Rs, but not RyRs, on the ER membrane blocked refilling (2016). Notably, IP3R inhibition induces lysosome dysfunction and LSD-like phenotypes in cells (2016). The inhibition of refilling by IP3R inhibitors argues against the possibility of GCaMP signaling being mediated by ER Ca^2+^ release and then amplified by lysosomal Ca^2+^ (Kilpatrick et al., 2016a, b). The inference that Ca^2+^ store refilling is mediated by IP3Rs, but not RyRs, suggests that ER Ca^2+^ release induced by lysosomal Ca^2+^ release may not operate through Ca^2+^-induced Ca^2+^ release because RyRs are better suited for this role than IP3Rs. Consistently, when Ca^2+^ levels in the lysosome lumen were measured with lysosome-targeted Fura-Dextran dye, it was shown that depleting ER Ca^2+^ or inhibiting IP3Rs also blocked refilling (2016). In another recently published independent study in which lysosomal Ca^2+^ was monitored with a pH-insensitive aequorin-based probe fused with a cathepsin protein, lysosomal Ca^2+^ was not refilled if SERCA activity was inhibited (Ronco et al., 2015). Taken together, these studies employing both juxta- and intra-lysosomal Ca^2+^ sensors/dyes suggest that lysosome stores are refilled with Ca^2+^ from the ER, independent of lysosomal pH.

## ER-dependent three-step model of lysosomal Ca^2+^ refilling

**Figure 2:**
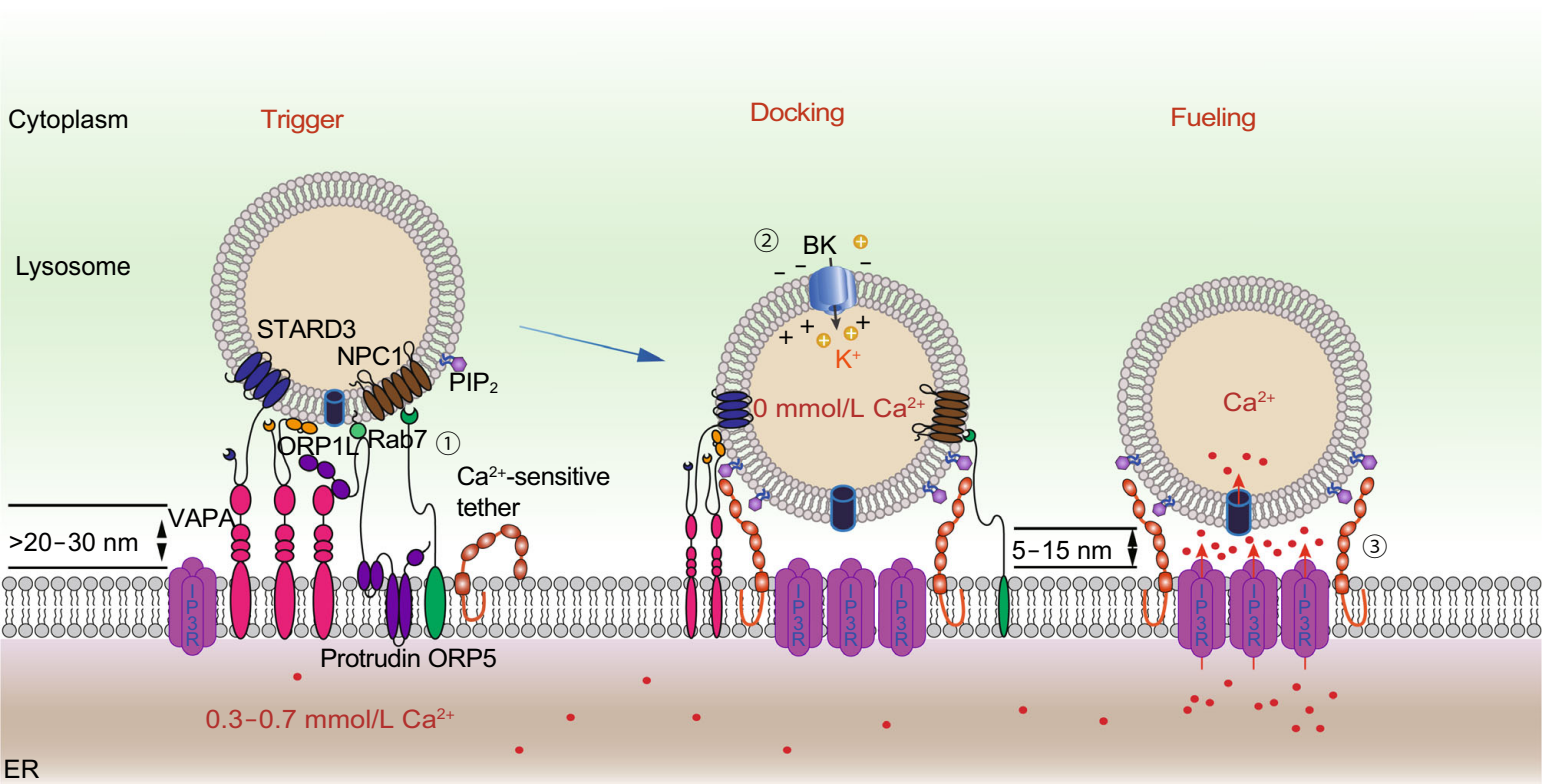
**A three-step working model of lysosomal refilling**. Lysosome Ca^2+^ stores are depleted upon cellular stimulation triggering lysosomal Ca^2+^ release. An increase in juxta-lysosomal [Ca^2+^]_Cyt_ or a decrease in [Ca^2+^]_Ly_ triggers refilling. In the docking step, MCSs are formed by both constitutive tethers, including endolysosome-localized ORP1L, STARD3, Protrudin, NPC1, and ER-localized ORP5, and VAP ([vesicle-associated membrane protein]-associated ER protein), as well as by putative Ca^2+^-sensitive tethers (e.g., E-syt1). In the fueling step, ER and lysosomal membranes are brought closer (within 5 nm). Meanwhile, both IP3Rs and putative uptake channel/transporters are enriched in ER-lysosome MCSs. Ca^2+^ released from lysosomes induces a conformational change of E-syt1-like protein on ER membranes, which in turn triggers the binding of E-syt1 with PI(4,5)P_2_, or other phosphoinositide, on lysosomal membranes, creating a functional ER-lysosome contact site for refilling. Ca^2+^ is then released from the ER via IP3Rs, causing a steep Ca^2+^ gradient that drives the influx of Ca^2+^ via an unidentified lysosomal uptake channel/transporter

A possible critical role of the ER in lysosomal Ca^2+^ refilling is reinforced by the structurally intimate localization of the ER and lysosomes at ER-lysosome MCSs (Phillips and Voeltz, 2016). An interesting testable hypothesis is that lysosomal Ca^2+^ refilling may be triggered directly by lysosomal Ca^2+^ release and may be dependent on ER-lysosome interactions that are dynamically regulated by lysosomal Ca^2+^. A similar model was proposed to explain ER store refilling wherein ER Ca^2+^ release triggers ER-PM functional coupling via STIM and Orai proteins (Saheki and De Camilli, 2017). Hence, lysosomal refilling may be a regulated, three-step process (Fig. 2): 1) triggering, by increased peri-lysosomal Ca^2+^ and/or decreased [Ca^2+^ ]_LY_; 2) docking, involving the formation of ER-lysosome MCSs; and 3) fueling, wherein Ca^2+^ is transported from the ER to lysosomes through functional ER-lysosome MCSs.

### Docking: formation of ER-lysosome MCSs

MCSs are close (typically <30 nm) appositions with tethering, but not fusion of membranes between organelles (Phillips and Voeltz, 2016). That is, they provide physical platforms for material exchange between organelles via a direct, non-fusion mechanism (English and Voeltz, 2013; Phillips and Voeltz, 2016; Saheki and De Camilli, 2017). Although ER-lysosome MCSs are well documented (Phillips and Voeltz, 2016), their functional significance is not clear. In comparison, ample evidence supports the involvement of ER-PM and ER-mitochondrial MCSs in Ca^2+^ exchange. In ER-PM MCSs, STIM and Orai proteins are concentrated (Saheki and De Camilli, 2017), and the oligomerization of ER-localized STIM1 activates Ca^2+^ influx via PM-localized Orai1 channels, thereby enabling refilling of ER Ca^2+^ stores (Saheki and De Camilli, 2017). Likewise, in ER-mitochondria MCSs, a protein complex is formed by mitochondrion-outer-membrane-localized VDAC channels, ER-localized IP3Rs, and the tethering protein Grp75, facilitating Ca^2+^ uptake from the ER to mitochondria (De Stefani et al., 2016; Krols et al., 2016; Phillips and Voeltz, 2016). Generally speaking, the short (<30 nm) distance between the ER and lysosomal membranes in MCSs should enable quiescent ER-to-lysosome Ca^2+^ transport without causing global [Ca^2+^]_Cyt_ increases.

ER-lysosome MCS formation requires several tethering proteins to keep the two opposing membranes in apposition, including oxysterol-binding protein-related protein 1L (ORP1L) (Rocha et al., 2009), protrudin (Raiborg et al., 2016), stAR-related lipid transfer protein 3 (STARD3) and oxysterol-binding protein-related protein 5 (ORP5)/Niemann-Pick C1 protein (NPC1) (Du et al., 2011; van der Kant and Neefjes, 2014; Phillips and Voeltz, 2016) (Fig. 2). Given that lysosome Ca^2+^ refilling requires peri-lysosomal increases in [Ca^2+^]_Cyt,_ it is likely that at least some tethering events may be regulated by peri-lysosomal Ca^2+^ (Wang et al., 2017). MCS gaps could be reduced, from 20–30 nm to within 5–15 nm, to provide a functional conformation highly amenable to Ca^2+^ exchange (Phillips and Voeltz, 2016). Several E-Syts (extended synaptotagmin-like proteins) have been confirmed to act as tethers at ER-PM MCSs (Min et al., 2007; Giordano et al., 2013). E-Syts have an N-terminal β-hairpin embedded in the ER membrane and multiple C2 domains in the C-terminal, which contains binding sites for both Ca^2+^ and phospholipids (Min et al., 2007; Giordano et al., 2013). Similar Ca^2+^ sensor proteins may play equivalent roles in ER-lysosome MCSs. Both PI(4,5)P_2_ and PI(3,5)P_2_ have been observed in lysosomes (Xu and Ren, 2015). Thus, upon Ca^2+^ release from lysosomes, a Ca^2+^- and phospholipid-dependent interaction between the two membranes may, in addition to the pre-existing tethers, help bring ER and lysosomes even closer, further supporting the functionality of ER-lysosome MCSs (Fig. 2).

### Fueling: Ca^2+^ transport in ER-lysosome ECS

After docking, a steep gradient between Ca^2+^-loaded ER and Ca^2+^-depleted lysosomes can drive the transfer of Ca^2+^ from the ER to lysosomes. This process appears to involve the coordinated actions of IP3R-mediated Ca^2+^ release from the ER and lysosomal Ca^2+^ uptake via a putative uptake channel or transporter (Fig. 2). Although accumulation of IP3Rs through lateral diffusion in ER-lysosome MCSs has not yet been demonstrated directly, constitutive Ca^2+^ release mediated by local enriched IP3Rs has been reported on ER-mitochondrial MCSs (Szabadkai et al., 2006; Cardenas et al., 2010). Because IP3Rs are constitutively active, refilling is plausible given very high local Ca^2+^ concentrations in MCSs, without a widespread Ca^2+^ release (Rizzuto et al., 1998).

Theoretically, any Ca^2+^-permeable channel, exchanger or pump could mediate lysosomal Ca^2+^ uptake. The slow nature of the refilling process suggests that it involves either a low affinity Ca^2+^ transporter or a rectifying Ca^2+^ channel (2016). Interestingly, low-affinity (mmol/L range) Ca^2+^ transporters have been observed in isolated lysosomes (Lemons and Thoene, 1991). It is also possible that a putative VDAC-like channel in lysosomes might mediate the Ca^2+^ uptake (van der Kant and Neefjes, 2014).

### Regulation of lysosomal Ca^2+^ refilling

Lysosomal ∆ψ appears to play a role in refilling (Wang et al., 2017). So-called big potassium (BK) channels, which regulate ∆ψ in excitable cells, exhibit functional expression in lysosomes (Cao et al., 2015a, b; Wang et al., 2017). Hence, Ca^2+^ activation of voltage-dependent, K^+^-selective conductance via BK channels may facilitate lysosomal Ca^2+^ release and refilling (Cao et al., 2015a, b; Wang et al., 2017). Hence, although there is no direct evidence, it is conceivable that lysosomal ∆ψ could affect refilling directly or indirectly. For example, it was reported recently that membrane potential can affect phosphoinositide dynamics (Zhou et al., 2015); and phosphoinositides are known to influence the interaction of lysosomes with other organelles, including peroxisomes and the ER (Chu et al., 2015). Hence, lysosomal Ca^2+^ refilling may require the action of multiple Ca^2+^ effectors in the triggering step, such as lysosome-localized BK channels, ER-localized IP3Rs and ER-localized E-Syt-like proteins (Fig. 2).

## Diseases associated with lysosomal Ca^2+^ store defects

Dys-regulation of lysosome Ca^2+^ homeostasis causes LSDs and lysosome-related diseases. Notably, ML-IV is associated with impaired lysosomal Ca^2+^ release (Kiselyov et al., 2010). Additionally, lysosomal Ca^2+^ stores have been reported to be reduced in Niemann-Pick, type C cells (Lloyd-Evans et al., 2008). Moreover, in Niemann-Pick, type C cells (containing *NPC1* mutation), TRPML1 activity was found to be inhibited by cholesterol accumulation in lysosomes, and increasing TRPML1 activity alleviated lysosomal storage in these cells (Shen et al., 2012). Indeed, compromised TRPML1 activity has been implicated in a number of LSDs (De Leo et al., 2016; Zhong et al., 2017). Additionally, lysosomal Ca^2+^ store defects are implicated in common neurodegenerative diseases, such as familial Alzheimer’s disease (Coen et al., 2012; Lee et al., 2015). A recent report showed that Parkinson disease patients’ cells with *GBA1* or *LRRK2* mutations (common risk factors of the disease) exhibit dysregulated lysosomal Ca^2+^ stores (Hockey et al., 2015; Kilpatrick et al., 2016a, b). It would not be surprising if more lysosome-related diseases associated with defects in lysosomal Ca^2+^ signaling are discovered (for detailed reviews, please see (Kiselyov et al., 2010; Morgan et al., 2011; Feng and Yang, 2016).

## Other vesicular Ca^2+^ stores

There are various types of common cellular vesicles with Ca^2+^-regulated membrane trafficking, including early endosomes, recycling endosomes, phagosomes, autophagosomes, secretory vesicles and peroxisomes (Fig. 1), as well as additional Ca^2+^-regulated vesicles in specialized cell types, such as synaptic vesicles in neurons, melanosomes in melanocytes (Bellono and Oancea, 2014), tubulovesicles in parietal cells (Sahoo et al., 2017) and lytic granules in cytotoxic T-cells (Clark and Griffiths, 2003; Patel and Cai, 2015). These organelles are capable of storing and releasing Ca^2+^ and, thus, are also considered to be small Ca^2+^ stores (Fig. 1).

Studying Ca^2+^ channels in these atypical Ca^2+^ stores remains a challenge. Tubulovesicles have been long proposed as vesicular Ca^2+^ stores that undergo exocytosis, bringing H^+^/K^+^-ATPase proton pumps to the apical membranes of parietal cells upon histamine stimulation. Recently, Sahoo et al. (2017) demonstrated that TRPML1 is localized on the tubulovesicular membranes of parietal cells, and that upon histamine-protein kinase A pathway activation, Ca^2+^ is released, inducing tubulovesicle exocytosis. Whereas TRPML1 knockout mice and ML-IV patients are achlorhydric (lacking acid secretion), TRPML1 overexpressing transgenic exhibits constitutive acid secretion (Sahoo et al., 2017). This work established TRPML1-mediated Ca^2+^ release as a missing link between histamine-protein kinase A signaling and tubulovesicle exocytosis (Sahoo et al., 2017). However, the refilling mechanisms for these vesicles are completely unknown.

## Conclusions and future directions

Although it is supposed that the ER may refill lysosomal Ca^2+^ stores, the underlying mechanisms of such refilling have not been delineated. Experiments showing that inhibition of ER Ca^2+^ and, more specifically, IP3Rs can abolish lysosomal store refilling support the notion that the ER may be the primary source of lysosomal Ca^2+^ under physiological conditions. Hence, small secondary stores, including lysosomes, may acquire Ca^2+^ from large primary stores, which in turn are refilled by extracellular Ca^2+^. In the near future, we expect to see advancements in the following areas:

- Identification of more regulators of ER-lysosome MCS formation.
- Revelation of lysosomal ∆ψ and Ca^2+^ roles in the regulation of ER-lysosome MCS formation.
- Development of organelle-targeted voltage dyes and luminal Ca^2+^ sensors enabling the study of ER-lysosome MCS dynamics with super-resolution live imaging.
- Identification of low-affinity uptake channels or transporters in the lysosome.
- Discovery of additional small, vesicular and mobile Ca^2+^ stores, together with their Ca^2+^ release channels and Ca^2+^ uptake transporters in these vesicles.
